# An international mixed methods study to develop a new preference-based measure for women with breast cancer: the BREAST-Q Utility module

**DOI:** 10.1186/s12905-020-01125-z

**Published:** 2021-01-06

**Authors:** Manraj N. Kaur, Anne F. Klassen, Feng Xie, Louise Bordeleau, Toni Zhong, Stefan J. Cano, Elena Tsangaris, Trisia Breitkopf, Ayse Kuspinar, Andrea L. Pusic

**Affiliations:** 1 McMaster University, 3N27, 1280 Main Street W, Hamilton, ON L8N 3Z5 Canada; 2 McMaster University, CRL-223, 1280 Main Street W, Hamilton, ON L8N 3Z5 Canada; 3 Juravinski Cancer Center, Room 3-17, 699 Concession Street, Hamilton, ON L8V 5C2 Canada; 4 Toronto General Hospital, Norman Urquhart Wing, Toronto, ON 8N-871M5G 2C4 Canada; 5 Modus Outcomes, Suite 210b, Spirella Building, Letchworth Garden City, SG6 4ET UK; 6 Brigham and Women’s Hospital, 75 Francis S, Boston, MA 02116 USA; 7 McMaster University, Room 435, 1400 Main Street West, Hamilton, ON L8S 1C7 Canada

**Keywords:** Health utility, Preference-based measure, Breast cancer, Qualitative, Patient-reported outcomes, Economic evaluation, Cost-effectiveness, Cost-utility, Interviews

## Abstract

**Background:**

Generic preference-based measures (PBM), though commonly used, may not be optimal for use in economic evaluations of breast cancer interventions. No breast cancer-specific PBM currently exists, and the generic PBMs fail to capture the unique concerns of women with breast cancer (e.g., body image, appearance, treatment-specific adverse effects). Hence, the objective of this study was to develop a breast cancer-specific PBM, the BREAST-Q Utility module.

**Methods:**

Women diagnosed with breast cancer (stage 0–4, any treatment) were recruited from two tertiary hospitals in Canada and one in the US. The study followed an exploratory sequential mixed methods approach, whereby semi-structured interviews were conducted and at the end of the interview, participants were asked to list their top five health-related quality of life (HRQOL) concerns and to rate the importance of each item on the BREAST-Q. Interviews were audio-recorded, transcribed verbatim, and coded. Constant comparison was used to refine the codes and develop a conceptual framework. Qualitative and quantitative data were triangulated to develop the content of the Utility module  that was refined through 2 rounds of cognitive debriefing interviews with women diagnosed with breast cancer and feedback from experts.

**Results:**

Interviews were conducted with 57 women aged 55 ± 10 years. A conceptual framework was developed from 3948 unique codes specific to breasts, arms, abdomen, and cancer experience. Five top-level domains were HRQOL (i.e., physical, psychological, social, and sexual well-being) and appearance. Data from the interviews, top 5 HRQOL concerns, and BREAST-Q item ratings were used to inform dimensions for inclusion in the Utility module. Feedback from women with breast cancer (N = 9) and a multidisciplinary group of experts (N = 27) was used to refine the module. The field-test version of the HSCS consists of 10 unique dimensions. Each dimension is measured with 1 or 2 candidate items that have 4–5 response levels each.

**Conclusion:**

The field-test version of the BREAST-Q Utility module was derived from extensive patient and expert input. This comprehensive approach ensured that the content of the Utility module is relevant, comprehensive, and includes concerns that matter the most to women with breast cancer.

## Background

Breast cancer is the most common cancer and the second leading cause of cancer death globally [1]. In the United States, an estimated 276,480 new cases of invasive and 48,530 new cases of non-invasive breast cancer will be diagnosed in 2020 [1, 2]. Fortunately, breast cancer incidence rates in developed countries have been in decline since 2000 due to improvement in early diagnosis and advancement in therapy [1, 3]. Consequently, the number of breast cancer survivors is on the rise, with more than 3.1 million breast cancer survivors in the United States [2]. As survival increases, the focus of breast cancer interventions has shifted from survival to include improvements in health-related quality of life (HRQOL).

HRQOL is defined as the subjective perception of the impact of disease or its treatment(s) on an individuals’ physical, psychological, and social well-being [4]. HRQOL data provide unique information from patients’ perspectives on the persistent or late-onset effects of cancer treatments [5–7]. As such, data about HRQOL can be used to improve how breast cancer care is planned, organized, and delivered. A common approach to collecting HRQOL data is by means of patient-reported outcome measures (PROMs). PROMs tend to be either generic that capture the core dimensions of health across conditions, or condition-specific. Another type of PROM generates a profile of dimension scores or a single index that is based on either a summation of item scores with preference weights obtained from patients or the general public (known as preference-based measure (PBM) or multi-attribute utility measure). The index (or utility) value obtained from a PBM can be used to calculate quality-adjusted life-years, which is the metric of choice in economic evaluation of healthcare interventions [8].

In breast cancer, due to the lack of a condition-specific PBM (CSPBM), generic measures such as the EQ-5D [9, 10], the Short Form-6D (SF-6D) [11, 12], and the Finnish 15D [13, 14] are frequently used. Research has shown that generic PBMs may fail to evaluate outcomes relevant to the specific patient group, and hence, over- or under-estimate the cost-effectiveness of the interventions examined. In breast cancer, generic PBMs do not include the unique concerns of women, such as breast appearance, body image, or sexual well-being. Hence, the objective of this study was to develop the descriptive health state classification system for a new breast cancer-specific PBM module of the BREAST-Q [15], called the BREAST-Q Utility module.

## Methods

**Fig. 1:**
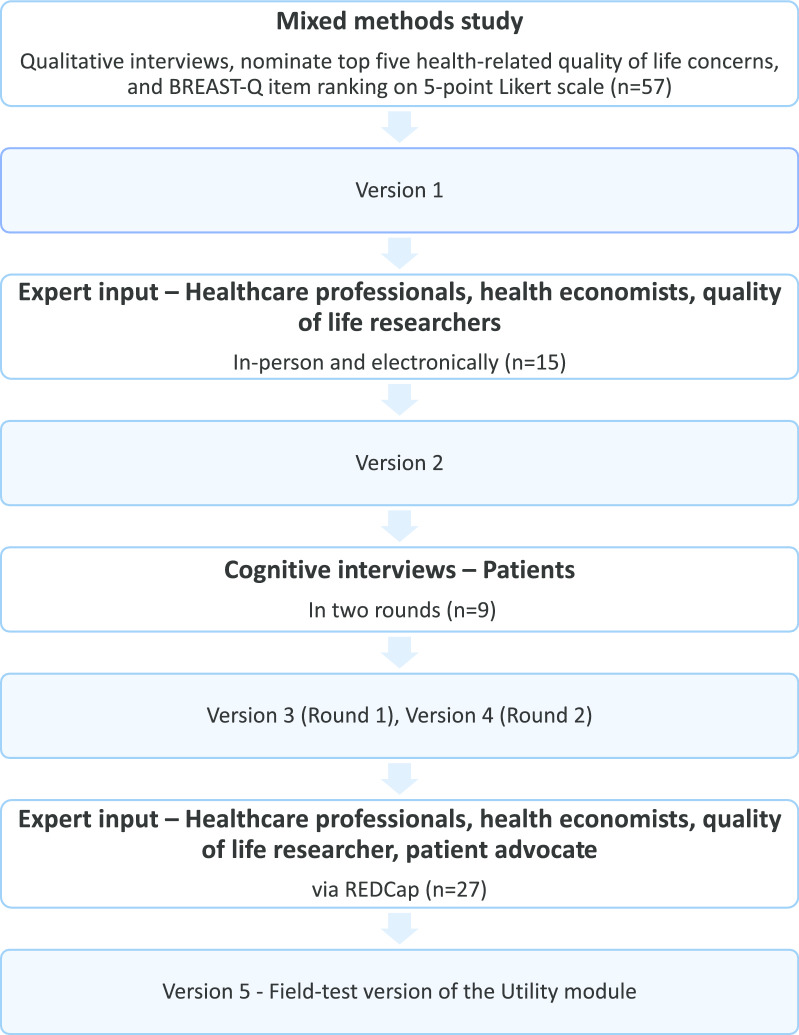
An overview of the steps used in the development of the BREAST-Q Utility module

The development of the BREAST-Q Utility module adhered to recommended methods for PROM instrument development [16–21]. The study followed a mixed methods approach using an exploratory sequential study design [22]. Figure 1 shows the steps taken to develop this new PBM. The study protocol is published elsewhere [23].

### Study setting and recruitment

Ethics board approval was obtained at three sites prior to recruitment: Juravinski Cancer Center -Hamilton and Toronto General Hospital (TGH), Ontario, Canada and Memorial Sloan Kettering Cancer Center (MSK), New York, United States. Purposive sampling was used to recruit English-speaking women with a confirmed diagnosis of breast cancer who varied by age (18 years and older), stage of breast cancer, and type of treatments. We excluded anyone seeking prophylactic or diagnostic interventions for breast cancer.

Eligible patients were invited to participate either during hospital visits or by phone by a member of the clinical team within their circle of care. Patients who expressed interest in participation were contacted by a member of the research team who described the study procedures and obtained consent. The interview was scheduled at a time and location convenient to the participant.

### Qualitative phase

We used an applied qualitative health research approach known as interpretive description. This inductive approach was inspired by grounded theory, naturalistic inquiry, ethnography, and phenomenology [24]. Interpretive description allows healthcare professionals to gain new insights from the clinical field, while taking into consideration existing knowledge regarding the clinical phenomenon [25].

#### Conceptual framework

The conceptual framework of the BREAST-Q was used to inform the study interview guide [15]. The BREAST-Q is a PROM designed to measure breast cancer surgery outcomes (breast-conserving therapy (BCT), mastectomy, and reconstruction). The BREAST-Q conceptual framework contains domains that are meaningful and relevant to women with breast cancer and was developed from extensive patient and expert input [26].

#### Data collection

Semi-structured interviews were conducted by two experienced qualitative researchers. Probes were used to elicit in-depth information about HRQOL domain(s). At the end of the interview, women were asked to nominate the top five concerns most important to their experience of breast cancer and its treatment(s). The interviews were audio-recorded and transcribed verbatim.

#### Analysis

Data were analyzed concurrent to interviews to allow new topics to be added to the interview guide for probing in subsequent interviews. Transcripts were coded using a combination of inductive (new codes) and deductive (application of existing BREAST-Q codes) approaches. Constant comparison was used to develop a codebook and refine codes. Top-level codes were kept broad to prevent pre-mature redundancy of concepts elicited during interviews. The conceptual framework was refined throughout the study. Interviews were conducted until researchers felt redundancy was achieved at the level of minor themes. The data on the top five HRQOL concerns were summarized descriptively.

#### Credibility

To establish credibility, the transcripts were coded by two experienced independent researchers who established consensus through discussion. The codebook and conceptual framework was reviewed by a senior author (AK), who provided ongoing feedback on the quality of the interviews, interview questions, and probes. The concepts elicited during the interviews were confirmed in subsequent interviews. The results of the data analysis were reviewed with research team members over the course of the study.

### Quantitative phase

To understand the importance of current BREAST-Q scales’ content to the breast cancer experience, women were asked to complete four scales (i.e., Satisfaction with Breasts and Psychosocial, Sexual, and Physical Well-being) within the most appropriate BREAST-Q Version 2.0 module [27] based on their surgical treatment (BCT, mastectomy, or reconstruction).

#### Data collection

At the end of the interview, women were asked to indicate how important (not important, slightly important, moderately important, important, and very important) each item on the BREAST-Q scale was to them. Completion of the BREAST-Q was on paper for in-person interviews or electronically for telephone interviews.

#### Analysis

BREAST-Q data were entered into IBM© SPSS Statistics Version 25. Using descriptive statistics, the BREAST-Q item rankings were summarized to identify the highest scoring items.

### Developing the BREAST-Q Utility module

The qualitative data, top five HRQOL concerns, and BREAST-Q item ranking exercise were triangulated to develop items for inclusion in the BREAST-Q Utility module. We followed PROM development principles [19, 20]: (1) domains should be relevant to the patient experience, (2) avoid negatively worded and double-barrelled items, (3) items should be easy to understand and not use slang or technical terms, (4) item wording should be easy to translate, (5) items and response options should retain participants’ words where possible, and (6) items should measure concepts that are likely to change with treatment or over time to enhance responsiveness. The qualitative data informed the choice of response options (e.g., severity versus frequency). Five response options were used to capture the range of health states experienced while reducing cognitive burden.

Feedback on a draft of the content of the Utility module and the wording of the instructions, items, and response options was obtained during a one-day, in-person meeting with quality of life researchers, healthcare professionals (breast surgeons, nurse), and a health economist. Feedback was also obtained through email from an international group of oncologists (medical, radiation, and surgical) and one psychometrician known to the investigators.

### Refining the BREAST-Q Utility module

Input from patients and healthcare professionals (HCPs) was used to establish content validity of the BREAST-Q Utility module.

#### Patient input

Women who took part in a qualitative interview and newly recruited participants were invited to take part in a cognitive interview to ensure that the content of the BREAST-Q Utility module was relevant, comprehensive, and comprehendible [18, 20]. Experienced interviewers used the “think aloud” technique [28–30] to obtain feedback on the instructions, items, and response options. The interviews were conducted in-person or over telephone, audio-recorded, and transcribed verbatim. Data were analyzed concurrently by one researcher and checked by another independent researcher using line-by-line coding. The Utility module was revised between two rounds of interviews. Interviews continued until no further changes were recommended by the three consecutive participants at the level of the items.

#### Expert input

An international multi-disciplinary group of experts was identified through the research team’s professional network and invited to review the Utility module using REDCap [31]. Feedback was sought on the wording of the instructions, items, and response options. The experts were also asked to rate the importance of the items on a 5-point Likert scale and to identify items that were missing. One reminder email was sent two weeks later. The expert feedback was examined descriptively, and the Utility module was revised.

## Results

A total of 57 qualitative interviews were conducted between January 2017 and June 2018. Interviews lasted 80 ± 34 min (range 30–162 min). The mean age of the sample was 55 ± 10 years (range, 22–75 years). Participant characteristics are shown in Table 1.

**Table 1:** Demographic and clinical characteristics of the sample

	Qualitative interviews N = 57		Cognitive interviews N = 9	
Characteristic	N	%	N	%
*Site of recruitment*				
Canada—JCC	22	39	6	67
Canada—TGH	21	37	3	33
United States—MSK	14	25	0	0
*Stage of breast cancer*				
Stage 0	9	16	1	11
Stage 1	15	26	2	22
Stage 2	20	35	5	56
Stage 3	10	18	1	11
Stage 4	3	5	0	0
*Age in years*				
Young adult (18–39)	2	4	0	0
Middle-aged adult (40–59)	39	68	6	67
Old adult (60 and above)	16	28	3	33
*Race/ethnicity*				
White	45	79	7	78
Black or African American	2	4	1	11
Asian	5	9	1	11
Other	5	9	0	0
*BMI category*				
Underweight—< 18.5	2	4	1	11
Normal—18.5 to 24.9	21	37	2	22
Overweight—25 to 29.9	24	42	4	44
Obese—30 and higher	10	18	2	22
*Marital status*				
Married/living common law	43	75	8	89
Single, never married	4	7	0	0
Divorced/separated/widowed	10	18	1	11
*Employment*				
Employed, full-time	24	42	3	33
Employed, part-time	12	21	3	33
Unemployed	2	4	0	0
Homemaker	3	5	0	0
Sick leave/disabled	3	5	0	0
Retired	11	19	1	11
Other	2	4	2	22
*Total annual household income (previous year)*				
0–25,000	5	9	0	0
25,000–50,000	5	9	0	0
50,000–75,000	8	14	2	22
> 75,000	31	54	7	78
Prefer not to say	8	14	0	0
*Education*				
High school graduate or equivalent	10	18	2	22
Some college/university (less than 4 years)	13	23	2	22
College/university (4-year bachelor’s degree)	28	49	4	44
Postgraduate degree (e.g., Masters, Doctorate)	6	11	1	11
*Type of (neo)adjuvant treatment*				
Chemotherapy	37	65	7	78
Radiation	35	61	7	78
Hormone replacement therapy	36	63	7	78
Targeted therapy (HER2)	7	12	0	0
*Type of cancer surgery*				
Breast conserving therapy	9	16	2	22
Mastectomy—Unilateral	24	42	5	56
Mastectomy—Bilateral	23	40	2	22
None	1	2	0	0
*Reconstruction*	N = 47		N = 7	
Yes	36	77	4	
No	11	23	3	
*Type of reconstruction*	N = 36		N = 4	
Autologous	26	72	2	50
Implant	10	28	2	50
*Laterality*				
Unilateral	18	50	2	50
Bilateral	18	50	2	50
*Timing of reconstruction*				
Immediate	21	58	2	50
Delayed	6	17	2	50
Not available	9	25	0	0

JCC, Juravinski Cancer Center, TGH, Toronto General Hospital, MSK, Memorial Sloan Kettering Cancer Center, HER2, Human epidermal growth factor receptor

### Qualitative phase

**Fig. 2:**
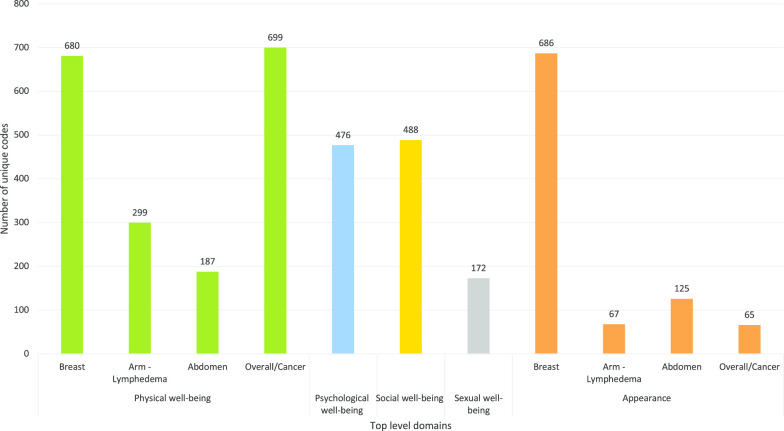
Item pool of the BREAST-Q Utility module

**Fig. 3:**
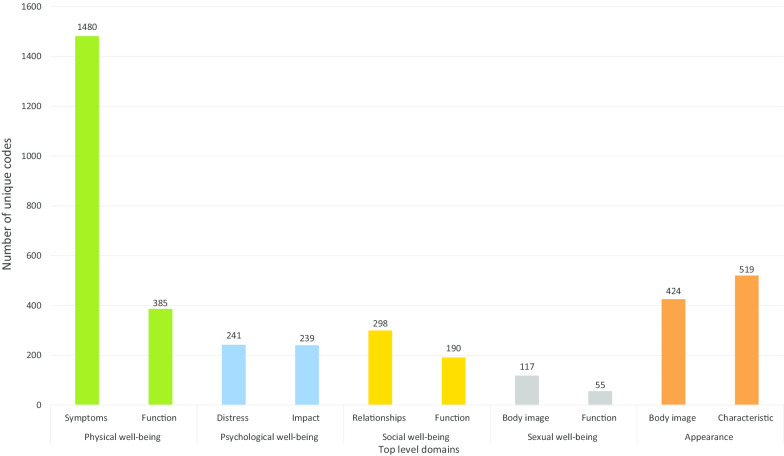
Conceptual framework of the BREAST-Q Utility module

The BREAST-Q Utility conceptual framework was developed from 3948 unique codes. Five top-level domains were identified: physical, psychological, social, and sexual well-being and appearance (Fig. 2). Figure 3 highlights the subdomains. The domains, sub-domains, and themes (or dimensions) are described in detail below.

#### Health-related quality of life

##### Physical well-being

This domain was used to capture symptoms and mobility-related issues specific to breast cancer surgery and the (neo)adjuvant treatments.

*Physical symptoms*

Fatigue

Breast cancer treatment-related fatigue was the most disabling symptom experienced by women actively receiving treatment or in survivorship. Women described the experience of fatigue as “tired”, “wiped out”, “lethargic”, “depleted of energy”, “drained”, and “exhausted”. Women also equated the feeling of fatigue with feeling physically weak or unwell. Frequent napping during the day and unrestful sleep at night were common complaints: “I had to have an afternoon nap during chemotherapy” and “I felt lousy in the night”.

Feeling tired interfered with women’s ability to do daily activities (e.g., “took longer), caring for self or dependents, participation in hobbies or social activities (“I missed church picnic as I felt really lousy”), and work (“I could not go back to work due to tiredness”), resulting in substantial distress. Women reported reduced interest in sexual activities (“too tired to have sex”) as a result of fatigue.

Pain and discomfort

Women who were undergoing treatment(s) commonly reported pain or discomfort that varied by type (dull, sharp, ache, shooting), intensity (mild, moderate, severe), frequency (constant, intermittent), and location. Pain due to breast cancer surgery was described as “dull ache”, “discomfort”, or occasionally as “sharp” or “electric”, and was worsened due to sleeping in certain positions (e.g., side-lying or prone) and movement of the arm(s). Women reported pain in the breast area, shoulder or arm, and due to wound care in the immediate postoperative period. For most women, pain interfered with sleep (“pain wakes me up at night”, “I cry out in pain in sleep”) and restricted their ability to participate in daily activities. Participants with abdomen-based reconstruction described feeling “discomfort”, “bloated”, or “tightness” in the abdomen area that was aggravated by sudden or sharp movements (e.g., coughing, straining for bowel movements).

Pain due to systemic therapy was described as “constant”, “deep”, “excruciating”, “sore everywhere”, “arthritic”, with or without morning stiffness, and was frequently experienced in lower extremity joints. This type of pain was often described as “debilitating”, and impacted sleep, mobility (e.g., walking, stairs), bed or chair transfers, and daily activities.

Breast sensation

Most women reported a lack of feeling (“numbness”) in their breast area (including axilla and in/around scar) for months following breast surgery. Many women reported their breast(s) intermittently feeling “hard”, “full”, “heavy”, “cooler than rest of the body”, and feeling “electric shocks”, “lightening”, or “firework-like sensations”. A small number of women experienced phantom symptoms on the surgical side, including “deep itch”, or “feeling of milk coming down”.

Peripheral neuropathy

Some participants who underwent systemic therapy experienced peripheral. The feelings of “numbness”, “tingling”, “pain”, or “pins and needles” was reported. Neuropathy in the hands was reported to interfere with fine motor tasks, such as holding a pen, buttoning a shirt, screwing or unscrewing jars, sewing, lifting a cup, or carrying or lifting grocery bags. Neuropathy in the feet caused pain or loss of balance and interfered with walking and physical activity.

Other symptoms

Less frequently described symptoms included altered taste, loss of appetite, nausea or vomiting, mouth sores, hot flashes, dry eyes, weight gain or loss, headaches, feeling lightheaded or dizzy, dyspnea, tachycardia, vaginal dryness or itching, and frequent urination. Some women described difficulty remembering things, and issues with recall, focus, or problem solving (“brain fog” or “chemo-brain”) during and for months following systemic therapy.

*Physical functioning*

Mobility and daily activities

Some women reported difficulty with moving or lifting the arm on the surgical side, especially in the immediate postoperative period. The reduced arm mobility interfered with personal hygiene (bathing, washing hair), self-care (applying makeup, styling hair, getting dressed), household chores (meal preparation, laundry), overhead activities (“I could not put stuff up in the high cupboard”), lifting objects (“I couldn’t lift or hold things for a long period”), driving, exercising, hobbies, and/or recreational activities. Women who were seeking or had undergone systemic therapies reported mobility issues due to pain and fatigue. This interfered with activities such as bed or chair transfers and doing stairs. Women with abdomen-based autologous reconstruction also reported difficulties with bed and chair transfers especially in the immediate post-operative period. Participants reported using accommodations such as hired help, bath chair, gait aids (walker, cane), and specialty shoes (for neuropathy).

Sleep

Women reported concerns with the amount and quality of sleep, including difficulties with falling asleep, staying asleep, and interrupted sleep often due to the side-effects such as nausea, hot flashes, pain, or discomfort. Suboptimal night sleep often resulted in daytime fatigue and women reported needing to nap during the day. Sleep was also affected in the post-operative period due to having to sleep in unfamiliar positions. A few women reported sleeping in a recliner chair or speciality bed during the postoperative period. Some women recovering from implant-based reconstruction felt discomfort or anxious about putting pressure on their implants in the prone position.

##### Psychological well-being

*Emotional distress*

Women described feeling anxious or worried about losing their breast(s), treatments and their side-effects, prognosis, cancer recurrence, and the impact of cancer and its treatment on their significant other and family members. Women described the off-treatment phase as particularly stressful as they no longer felt they were proactively preventing cancer recurrence (“not having chemotherapy makes me anxious”). Women reported feeling distress about new symptoms that appeared post-treatment (e.g., aches or pains) and about receiving test results.

Women described feeling angry, frustrated, disappointed, or irritated upon diagnosis. This feeling was often replaced with sadness (“depressed”, “upset”, “feel awful”, “overwhelmed”) about losing their breast(s), chemotherapy-induced alopecia, and inability to fully participate in daily activities during their treatment and the recovery period. All women with young children worried about the possibility of not experiencing life with their children. Some women reported dwelling on their diagnosis, effectiveness of the treatment(s), cancer recurrence, or late effects of treatments (e.g., cardiotoxicity).

*Positive impact*

A few women described their coping strategies and how they saw their diagnosis as an opportunity to restructure life, build personal connections, pursue new hobbies, or travel. Women were grateful for their support network, timely access to treatments, going into remission, and being alive (“I remember thinking I’ve lost a breast, but I haven’t lost my life”). Some women reported change in their outlook toward life and living with more gratitude and in the present.

##### Social well-being

This domain covered social issues in relation to the diagnosis, treatment, and survivorship phases. These codes were classified into social participation, isolation, and relationships.

*Social function*

Social participation

Women reported limitations in their ability to participate in their usual social roles, including caring for self and family, work, and community roles. Side-effects (e.g., pain, fatigue, neuropathy) of cancer treatments impacted women’s work-life into the recovery and survivorship phases. Some women modified their work responsibilities by asking for accommodations (“reduced the number of hours worked” or “took more breaks”) or discontinued employment (temporarily or permanently). Women also reported requiring assistance with childcare and help with household chores, resulting in an emotional or financial burden on the family.

Social isolation

Social isolation was described as necessary in the context of chemotherapy to avoid infection. Symptoms such as fatigue and body image-related issues (especially alopecia) interfered with the ability or choice to participate in social events. Treatment factors, such as the daily burden associated with radiation therapy, also prevented women from socializing with friends or family. Lack of participation in meaningful activities (work, or leisure) contributed to a sense of loneliness through the breast cancer experience.

*Relationships*

Provision of emotional, instrumental and informational support from others was central to women’s experience of breast cancer. Being driven to healthcare appointments, and help with housekeeping, meal preparation, and childcare was invaluable. Many women relied on visits and talking with family members to cope with their illness. Most women struggled with their new role as a dependent and worried about their partner taking on caregiving responsibilities. Given how common breast cancer is, participants leaned on relatives or friends who were diagnosed with breast cancer for information about treatments, side-effects, and remedies.

#### Sexual well-being

*Sexual self-image*

Changes in appearance due to breast cancer treatment affected women’s sexual self-image and sexual interactions. Most women reported feeling less attractive in intimate scenarios and reduced satisfaction due to pain or lack of sensation in the breast area. Some women were bothered by their partner looking at or touching their breast area and mentioned covering up during intimate scenarios.

*Sexual functioning*

Women who were sexually active expressed concerns due to fatigue, loss of libido, and vaginal dryness, itching or irritation, and dyspareunia, that impacted their ability to experience sexual pleasure. Women used terms such as “not as often”, “less frequent”, “not interested”, “non-existent”, or “lost intimacy” to describe their experience. Many survivors reported a persistent depressed mood or sadness and/or anxiety that lasted beyond the treatment phase secondary to fear of recurrence, impact on partner and family, and body image concerns. These factors affected some women’s ability to orgasm resulting in reduced sexual frequency. This was particularly relevant to younger women with breast cancer and single women who were seeking a partner.

#### Appearance

This domain captured women’s appraisal of their physical appearance. Subdomains were categorized into appearance of the breast, abdomen (autologous reconstruction using abdominal tissue), arm due to lymphedema, and overall appearance.

##### Breasts, breast area, and nipples

The appearance of the breast(s) or breast area before and after breast cancer surgery was the most frequently mentioned concept. Women appraised their breast area by describing the contour (“caved in,” “bulge”, “droopy”, “puckered”), symmetry (“closely matched”, “looked similar”, “one smaller than other”, or “one higher than the other”), shape (“concave”, “flat”, “hollow”, or “full”), size (“same”, “small”, or “bigger”), and ptosis (“hang”, or “droop”). Most women described the appearance of their breast in terms of how “natural” or “normal” they looked compared to before surgery and/or to other women. Women who had radiotherapy described changes to the skin of the breast area (“looks sunburnt”, “I have a permanent tan”).

##### Abdomen and belly button

For abdomen-based autologous reconstruction, women were bothered by a shift in the position and size of the belly button (“much larger than what I used to have”, “doesn’t look original”). The position and color of the abdomen scar were identified as issues that could be concealed with clothing. Some women were bothered by dog ears that were visible when clothed.

##### Arms-Lymphedema

Women with lymphedema described the appearance of their affected arm(s) in terms of the size (“bigger”), contour (“rounded”, or “full”), shape (“indented”), and color (“lighter”). Women mentioned challenges associated with concealing the arm (finding clothes that fit) and feeling self-conscious in public (“I wear long sleeves if I was going out for dinner”).

##### Overall appearance

All women who underwent chemotherapy experienced alopecia, and while some women were extremely bothered by alopecia, others perceived it as a temporary issue. Most women coped with alopecia by cutting their hair short prior to starting chemotherapy and by wearing a wig, scarf, baseball cap, or toque in pubic settings. Some women reported using makeup to conceal loss of eyebrows and eyelashes. A few women noted changes to their skin (“dry”, “pale”) and nails (“black”, “loss of nails”).

### Quantitative phase

#### Top five HRQOL concerns: patients

**Fig. 4:**
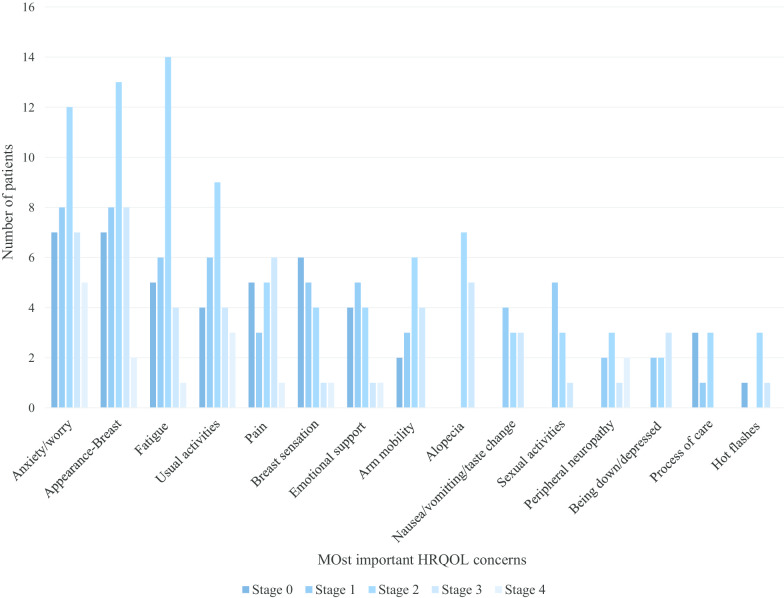
Top 15 domains by stage of breast cancer (n = 50)

The top HRQOL concerns by the stage of breast cancer are shown in Fig. 4. Overall, appearance of the breast(s), fatigue, cancer worry, impact on usual activities, and feeling anxious were the top five HRQOL concerns across all stages of breast cancer.

#### BREAST-Q- Item ratings: patients

Women consistently endorsed the following items to be important to their breast cancer experience across all three BREAST-Q modules: satisfaction with appearance (closely matched, feel natural, look in mirror unclothed), psychological well-being (confident, emotionally health, attractive), physical well-being (pain), sexual well-being (sexually attractive, confident sexually, sexually attractive when unclothed), and adverse effects of radiation (skin feeling dry, looking different). Women who had abdomen-based surgery endorsed difficulties sitting up, everyday activities, and discomfort in the abdomen area as the most important concerns.

### Selection of domains and dimensions within domains

The findings described above were used to develop the first draft of the BREAST-Q Utility module. This version measured 9 unique dimensions (i.e., 9 items)  with 4–5 response options each (Version 1). Based on the expert feedback, 3 new items were added, and the initial item measuring pain and unpleasant symptoms was split into two items. In addition, the instructions were modified to include a recall period and the wording of some of the items was modified. The revised version (Version 2) included 12 dimensions (14 items) with 4 response levels each.

### Refining the BREAST-Q Utility module

Cognitive interviews were conducted from October 2018 to April 2019. Interviews lasted 60 ± 14 min. Sample characteristics are shown in Table 1. Version 2 was shown to five women. Based on participant feedback, several items and the response options were revised, resulting in Version 3. This version was shown to four women and further revised resulting in Version 4. This version was developed into a REDCap survey. A total of 35 experts were invited to provide feedback out of which 27 responded (response rate, 68%). The experts included medical oncologists (n = 3), radiation oncologists (n = 3), breast surgeons (n = 15), health economics and/or outcomes researchers (n = 5), and a patient advocate. Experts were from the United States (n = 10), Canada (n = 7), the Netherlands (n = 4), Poland (n = 2) and Chile, Denmark, Italy, and United Kingdom (n = 1 each). The Utility module was revised based on the expert feedback, resulting in the field-test version of the Utility module (Version 5). Table 2 summarizes the item reduction and refinement steps.

**Table 2:** Summary of iterative revisions made to the BREAST-Q Utility module in Version 1–4

Version 1	Version 2—Expert feedback		Version 3—Round 1—Cognitive interviews—patients		Version 4—Round 2 Cognitive interviews—patients		Version 5 (Field-test version)—Expert feedback	
These questions ask about how your breast cancer and/or its treatment has affected you NOTE: If you had breast cancer surgery on both breasts, please answer thinking about the side (i.e., breast and/or arm) that causes you more difficulty or concern	REVISE	These questions ask about how your breast cancer and/or its treatment has affected you Please answer each question based on how you look and feel TODAY NOTE: If you had breast cancer surgery on both breasts, please answer thinking about the side (i.e., breast and/or arm) that causes you more difficulty or concern	RETAIN		RETAIN		REVISE	These questions ask about how your breast cancer and/or its treatment has affected you Please answer each question based on the PAST WEEK
How much do you experience pain and/or unpleasant sensations (e.g., pressure, tightness) in your breast area?	REVISE	How much bodily pain do you experience?	REVISE	How much pain do you experience?	RETAIN		REVISE	How much pain did you feel? Did pain interfere with your daily activities?
		Do you experience any unpleasant symptoms?	REVISE	Do you experience any unpleasant symptoms (e.g., nausea, hot flashes, tingling or numbness in hands or feet)?	RETAIN		REVISE	Did you experience any unpleasant symptoms? Did unpleasant symptoms interfere with your daily activities?
How much feeling do you have in your breast area?	RETAIN		RETAIN		RETAIN		REVISE	How much feeling (sensation) do you have in your breast area?
How self-conscious are you about how your breast area looks?	RETAIN		RETAIN		RETAIN		RETAIN	
How similar (closely matched) are your breasts?	RETAIN		REVISE	How similar are your breasts? NOTE: If you had a double mastectomy without breast reconstruction (i.e., you do not have breasts), please skip this question	REVISE	How similar (i.e., closely matched in size and shape) are your breasts?	REVISE	How closely matched (i.e., in size and shape) are your breasts?
How much distress (e.g., anxiety, worry, sadness) do you feel because of breast cancer?	RETAIN		REVISE	How much emotional distress do you experience?	REVISE	How much emotional distress (e.g., anxiety, worry) do you experience?	REVISE	How much emotional distress (e.g., anxiety, worry) did you experience? Did emotional distress (e.g., anxiety, worry) interfere with your daily activities?
How difficult is it for you to keep up with your usual roles and responsibilities (e.g., work, caring for others, social activities)?	RETAIN		REVISE	How difficult is it for you to keep up with your usual activities?	REVISE	How difficult is it for you to keep up with your usual activities (e.g., work, housework, caring for self or others, social life)?	REVISE	How difficult was it for you to keep up with your usual activities (e.g., work, housework, caring for self or others)? Was it difficult for you to keep up with your usual activities (e.g., work, housework, caring for self or others)?
How difficult is it for you to lift or move your arm?	RETAIN		RETAIN		RETAIN		REVISE	How difficult is it for you to lift or move your arm? Did difficulty lifting or moving your arm interfere with your daily activities? NOTE: If both of your arms were affected by breast cancer treatment, please answer thinking of the arm that causes you more difficulty or concern
How tired do you feel?	REVISE	How tired (i.e., fatigue) do you feel?	REVISE	How much fatigue do you feel?	RETAIN		REVISE	How tired did you feel? Did feeling tired interfere with your daily activities?
How difficult is it for you to do activities that use your abdomen (e.g., get out of bed, make bed)?	REVISE	Did you have breast reconstruction using your abdomen (i.e., TRAM or DIEP flap)? If yes, please answer the following question How difficult is it for you to do activities that use your abdomen (e.g., get out of bed, lift a heavy object)?	REVISE	Did you have breast reconstruction using your own skin and fat (i.e., abdomen, back, thigh)? If yes, please answer the following question Do you experience problems at the donor site where fat and skin were taken?	RETAIN		DROP	
	NEW	How much nausea do you experience?	RETAIN		RETAIN		REVISE	Did you experience any nausea? Did nausea interfere with your daily activities?
	NEW	How much neuropathy (i.e., tingling or numbness in your hands or feet) do you experience?	RETAIN		RETAIN		REVISE	Did you experience any neuropathy (i.e., tingling or numbness) in your hands or feet? Did neuropathy (i.e., tingling or numbness) in your hands or feet interfere with your daily activities?
	NEW	Did your breast cancer treatment include radiation therapy? If yes, please answer the following question How does the radiated skin on your breast area look (e.g., change in colour or texture)?	REVISE	How does your radiated breast area look and feel?	REVISE	How does your radiated breast area look and feel (e.g., colour, texture, tightness)?	REVISE	How does your radiated breast area look? How does your radiated breast area feel (e.g., texture, itchy)?

### Health state classification system

The field-test version (Version 5) of the BREAST-Q Utility module (Additional file 1) includes 10 unique dimensions: fatigue, pain, emotional distress, impact on usual activities, how the breasts match, feeling self-conscious about how breast(s) look, breast sensation, arm mobility, treatment-related unpleasant symptoms, nausea, peripheral neuropathy, and radiated skin changes. Each dimension is measured by one or two candidate items each with four or five response options. Response options for items asking about breast appearance, body image, and sensation were based on severity, while the response options for items asking about fatigue, pain and emotional distress included options to measure severity and interference with daily activities in order to test alternate ways of measuring these concepts. The final set of field-test items totalled 21.

## Discussion

We described the development of a breast cancer-specific PBM, the BREAST-Q Utility module, which we designed for women diagnosed with breast cancer of any stage and any combination of surgical or (neo)adjuvant treatments. To the best of our knowledge, this is the first breast cancer-specific PBM developed following recommendations and guidelines for the development of PROMs [16–20, 32]. The patient-driven “bottom-up” approach we took ensured that the content generated was grounded in the experiences of women with breast cancer and included the most relevant HRQOL domains from their perspective. A strength of this study is the diverse sample interviewed that included women who varied by pathological stage of disease and treatments (local and systemic) from private and public healthcare settings. This heterogeneity ensured that the 21 items are relevant to a wide range of women having breast cancer treatment.

Several approaches to develop condition-specific PBM have been described in the literature, including item reduction of existing PROMs using traditional or modern psychometric methods. Goodwin and Green [33] conducted a systematic review of literature of published condition-specific PBMs and found that out of the 51 published PBMs, 18 (35%) were developed de novo and the remaining used existing PROMs. Only two of the 18 de novo condition-specific PBMs were developed using data from different sources (e.g., qualitative interviews, expert opinion, literature review). Hence, our study adds to the literature on how to rigorously develop a condition-specific PBM that demonstrates content validity using a bottom-up approach.

The development of the BREAST-Q Utility module is timely as the International Society for Pharmacologic and Outcomes Research’s taskforce recommends the use of a PBM that is appropriate for a specific health condition and patient population, in addition to considering the requirements of the agency to which the economic evaluation will be submitted [34]. Hence, once completed, the BREAST-Q Utility module will be relevant in cases where the generic PBMs fail to include treatment outcomes important to women with breast cancer. Further, generic measures have been shown to have problems with floor and ceiling effects and sensitivity in certain patient populations [35, 36].

As such, utility values derived from a generic PBM in a trial may underestimate the benefits of an intervention. For example, in breast cancer, a trial assessing the treatment outcome of two different breast reconstruction approaches (implant versus autologous) that uses a generic PBM would be insensitive to measuring differences in breast appearance, body image, and breast(s) sensation. However, utility values derived from condition-specific PBM exclusively may overestimate the treatment benefit and not allow for comparability across health conditions and interventions. Since utility values derived from condition-specific PBMs are currently not accepted in base-case cost-effectiveness analysis by most of the international agencies, we recommend using the BREAST-Q Utility module alongside a generic PBM in economic evaluations of breast cancer interventions.

Our study adds to the growing body of evidence about the impact of breast cancer diagnosis and treatments on HRQOL. Consistent with the previous qualitative and quantitative studies, we found that the diagnosis of breast cancer and its treatments have a negative impact on breast appearance [15, 37], overall appearance [38], body image [39, 40], physical [41, 42], psychological [43–45], social [46–48] and sexual well-being [49, 50], and overall HRQOL [15, 51–54]. In addition to development of the BREAST-Q Utility module, the rich information we collected in our qualitative study has been used to develop some new scales and modules to measure concepts not covered by the BREAST-Q, including breast sensation [55] and arm lymphedema [56].

A limitation of our study is that the sample included only English-speaking women with breast cancer living in North America. Further, most participants were diagnosed with early stage breast cancer. As a result, domains that are relevant to middle to older-aged women with earlier stages of cancer may have been over-represented in the Utility module. With advancements in early diagnosis and prevention, most of the women in developed countries are diagnosed at early stages [2, 57] and hence, from a health technology assessment and policy perspective, this is where the BREAST-Q Utility module has the most relevance. To address this limitation, the expertise of a multidisciplinary sample of healthcare professionals with experience in caring for patients with breast cancer were included at various stages of scale refinement. In the next phase of the study, a large sample of breast cancer patients will be surveyed to examine response patterns and data quality and to identify patterns in responses by specific breast cancer subgroups (e.g., cancer stage, treatment types) and patient demographics (e.g. age).

### Conclusion

This paper describes the development of the new BREAST-Q Utility module using a mixed-methods approach and best practice guidelines for PROM development. The content of the BREAST-Q Utility module is grounded in extensive feedback from women diagnosed with breast cancer and healthcare professionals. The next phase of research will examine the pattern of responses and psychometric properties of the Utility module in a large sample of women with breast cancer, followed by a valuation survey to elicit utility weights for each dimension included in the module. Once developed, the BREAST-Q Utility module will be available for use in clinical research and in economic evaluations of breast cancer interventions through the Q-Portfolio webpage (www.qportfolio.org).

## Supplementary information

**Additional file 1.** Field-test version of the BREAST-Q Utility module.

## Data Availability

The datasets used and/or analysed during the current study are available from the corresponding author on reasonable request.

## Abbreviations

BCT: Breast Conserving Therapy
BMI: Body Mass Index
EQ-5D: EuroQol-5 Dimension
HCP: Healthcare Professional
HRQOL: Health-Related Quality of Life
MSK: Memorial Sloan Kettering Cancer Center
PBM: Preference-Based Measure
PROM: Patient-Reported Outcome Measure
SF-6D: Short Form-6 Dimension
TGH: Toronto General Hospital
US: United States
