# Supplementary material for: An international mixed methods study to develop a new preference-based measure for women with breast cancer: the BREAST-Q Utility module
Source: BMC Womens Health. 2021 Jan 6;21:8. doi: 10.1186/s12905-020-01125-z (PMC7789506; doi:10.1186/s12905-020-01125-z)
Supplement: Supplementary file 1 — Additional file 1. Field-test version of the BREAST-Q Utility module. [file 12905_2020_1125_MOESM1_ESM.docx]

**Additional file 1: Field-test version of the BREAST-Q Utility module**

***(NOTE: Once the field-test of the BREAST-Q Utility module is complete, the items will be reduced and refined as appropriate. This is NOT THE FINAL VERSION of the Utility module)***

**These questions ask about how your breast cancer and/or its treatment has affected you. Please answer each question based on the PAST WEEK.**

1. **How tired did you feel?**

I did NOT feel tired.

I felt a LITTLE tired.

I felt QUITE tired.

I felt VERY tired.

I felt EXTREMELY tired.

1. **Did feeling tired interfere with your daily activities?**

I did NOT feel tired.

I felt tired but it interfered with NONE of my daily activities.

I felt tired and it interfered with SOME of my daily activities.

I felt tired and it interfered with MOST of my daily activities.

I felt tired and it interfered with ALL of my daily activities.

1. **How much pain did you feel?**

I had NO pain.

I had MILD pain.

I had MODERATE pain.

I had SEVERE pain.

1. **Did pain interfere with your daily activities?**

I had NO pain.

I had pain but it interfered with NONE of my daily activities.

I had pain and it interfered with SOME of my daily activities.

I had pain and it interfered with MOST of my daily activities.

I had pain and it interfered with ALL of my daily activities.

1. **How much emotional distress (eg, anxiety, worry) did you experience?**

I experienced NO distress.

I experienced MILD distress.

I experienced MODERATE distress.

I experienced SEVERE distress.

1. **Did emotional distress (eg, anxiety, worry) interfere with your daily activities?**

I experienced NO distress.

I experienced distress but it interfered with NONE of my daily activities.

I experienced distress and it interfered with SOME of my daily activities.

I experienced distress and it interfered with MOST of my daily activities.

I experienced distress and it interfered with ALL of my daily activities.

1. **How difficult was it for you to keep up with your usual activities (eg, work, housework, caring for self or others)?**

It was NOT difficult.

It was a LITTLE difficult.

It was QUITE difficult.

It was VERY difficult.

It was EXTREMELY difficult.

1. **Was it difficult for you to keep up with your usual activities (eg, work, housework, caring for self or others)?**

It was NOT difficult for me to keep up with my usual activities.

It was difficult for me to keep up with SOME of my usual activities.

It was difficult for me to keep up with MOST of my usual activities.

It was difficult for me to keep up with ALL of my usual activities.

1. **How self-conscious were you about how your breast area looks?**

I was NOT self-conscious about my breast area.

I was a LITTLE self-conscious about my breast area.

I was QUITE self-conscious about my breast area.

I was VERY self-conscious about my breast area.
 I was EXTREMELY self-conscious about my breast area.

1. **How much feeling (sensation) do you have in your breast area?**

**NOTE: If you had breast cancer surgery on both breasts, please answer thinking about the breast that causes you more difficulty or concern.**

I have COMPLETE feeling in my breast area.

I have a LOT of feeling in my breast area.

I have SOME feeling in my breast area.

I have a LITTLE feeling in my breast area.

I have NO feeling in my breast area.

1. **How closely matched (ie, in size and shape) are your breasts?**

My breasts are closely matched (NOT different).

My breasts are a LITTLE different

My breasts are QUITE different.

My breasts are VERY different.

My breasts are EXTREMELY different.

1. **How difficult is it for you to lift or move your arm?**

**NOTE: If both of your arms were affected by breast cancer treatment, please answer thinking of the arm that causes you more difficulty or concern.**

It is NOT difficult for me to lift or move my arm.

It is a LITTLE difficult for me to life to move my arm.

It is QUITE difficult for me to lift or move my arm.

It is VERY difficult for me to lift or move my arm.

It is EXTREMELY difficult for me to lift or move my arm.

1. **Did difficulty lifting or moving your arm interfere with your daily activities?**

It was NOT difficult to lift or move my arm.

My arm was difficult to lift or move but it interfered with NONE of my daily activities.

My arm was difficult to lift or move and it interfered with SOME of my daily activities.

My arm was difficult to lift or move and it interfered with MOST of my daily activities.

My arm was difficult to lift or move and it interfered with ALL of my daily activities.

1. **Did you experience any unpleasant symptoms?**

I had NO unpleasant symptoms.

I had MILD unpleasant symptoms.

I had MODERATE unpleasant symptoms.

I had SEVERE unpleasant symptoms.

1. **Did unpleasant symptoms interfere with your daily activities?**

I had NO unpleasant symptoms.

I had unpleasant symptoms but they interfered with NONE of my daily activities.

I had unpleasant symptoms and they interfered with SOME of my daily activities.

I had unpleasant symptoms and they interfered with MOST of my daily activities.

I had unpleasant symptoms and they interfered with ALL of my daily activities.

1. **Did you experience any nausea?**

I had NO nausea.

I had MILD nausea.

I had MODERATE nausea.

I had SEVERE nausea.

1. **Did nausea interfere with your daily activities?**

I had NO nausea.

I had nausea but it interfered with NONE of my daily activities.

I had nausea and it interfered with SOME of my daily activities.

I had nausea and it interfered with MOST of my daily activities.

I had nausea and it interfered with ALL of my daily activities.

1. **Did you experience any neuropathy (ie, tingling or numbness) in your hands or feet?**

I have NO neuropathy.

I have MILD neuropathy.

I have MODERATE neuropathy.

I have SEVERE neuropathy.

1. **Did neuropathy (ie, tingling or numbness) in your hands or feet interfere with your daily activities?**

I have NO neuropathy.

I have neuropathy but it interfered with NONE of my daily activities.

I have neuropathy and it interfered with SOME of my daily activities.

I have neuropathy and it interfered with MOST of my daily activities.

I have neuropathy and it interfered with ALL of my daily activities.

______________________________________________________________________________

**Did your breast cancer treatment include radiation therapy? If yes, please answer the following question.**

1. **How does your radiated breast area look?**

**NOTE: If you had radiation on both breasts, please answer thinking about the breast that bothers you the most.**

My breast area looks the SAME as before radiation.

My breast area looks a LITTLE different than before radiation.

My breast area looks QUITE different than before radiation.

My breast area looks VERY different than before radiation.

My breast area looks EXTREMELY different than before radiation.

1. **How does your radiated breast area feel (eg, texture, itchy)?**

**NOTE: If you had radiation on both breasts, please answer thinking about the breast that bothers you the most.**

My breast area feels the SAME as before radiation.

My breast area feels a LITTLE different than before radiation.

My breast area feels QUITE different than before radiation.

My breast area feels VERY different than before radiation.

My breast area feels EXTREMELY different than before radiation.

**BREAST-Q^TM^ ©2009 Memorial Sloan-Kettering Cancer Center. All rights reserved.**

_____________________________________________________________________________
